# Genetics of Bone and Muscle Interactions in Humans

**DOI:** 10.1007/s11914-019-00505-1

**Published:** 2019-02-28

**Authors:** Katerina Trajanoska, Fernando Rivadeneira, Douglas P. Kiel, David Karasik

**Affiliations:** 1 Department of Internal Medicine, Erasmus MC, University Medical Center Rotterdam, Rotterdam, Netherlands; 2 Hebrew SeniorLife, Institute for Aging Research, Boston, MA USA; 3 Department of Medicine, Beth Israel Deaconess Medical Center and Harvard Medical School, Boston, MA USA; 4 Broad Institute of Harvard and Massachusetts Institute of Technology, Boston, MA USA; 5 Azrieli Faculty of Medicine, Bar-Ilan University, Safed, Israel

**Keywords:** Bone, Muscle, Osteoporosis, Sarcopenia, Genome-wide association study (GWAS), Pleiotropy

## Abstract

**Purpose of Review:**

To summarize the evidence from recent studies on the shared genetics between bone and muscle in humans.

**Recent Findings:**

Genome-wide association studies (GWAS) have successfully identified a multitude of loci influencing the variability of different bone or muscle parameters, with multiple loci overlapping between the traits. In addition, joint analyses of multiple correlated musculoskeletal traits (i.e., multivariate GWAS) have underscored several genes with possible pleiotropic effects on both bone and muscle including *MEF2C* and *SREBF1*. Notably, several of the proposed pleiotropic genes have been validated using human cells or animal models.

**Summary:**

It is clear that the study of pleiotropy may provide novel insights into disease pathophysiology potentially leading to the identification of new treatment strategies that simultaneously prevent or treat both osteoporosis and sarcopenia. However, the role of muscle factors (myokines) that stimulate bone metabolism, as well as osteokines that affect muscles, is in its earliest stage of understanding.

## Introduction

Osteoporosis and sarcopenia are common and costly comorbid diseases of aging, and there is an urgent need to prevent and treat both to reduce their associated morbidity and mortality [1••]. A burgeoning body of work shows that they share many common risk factors and biological pathways such as effects of growth hormone and inflammatory cytokines [2, 3]. Moreover, the common mesenchymal origin of bone and muscle cells underpins the tight link between these conditions from the early stages of embryonic development.

Muscle mass and function are important determinants of skeletal growth and bone mass accrual in growing humans. This adaption of bone tissue to loading follows the principles of Frost’s mechanostat theory, i.e., bone growth and bone loss are stimulated by the muscle forces/loads acting upon bone surfaces [4]. In addition to their mechanical interaction, bone and muscle are jointly regulated by hormones, and inextricably linked genetically and molecularly [5]. However, the latter interactions are difficult to observe and measure; thus, their roles are less well recognized. The recent rise of new technologies (arising from genetics, molecular biology etc.) has shed new light on the genetic and molecular interplay between bone and muscle; thus, our understanding of these interactions has evolved over time. In the past few years, studies have endeavored to (1) disentangle the intricate molecular mechanisms that lead to osteoporosis and sarcopenia and (2) develop new treatment strategies by pinpointing drug targets common to both conditions.

The aim of this review is to summarize the evidence from current studies on the shared genetics between bone and muscle in humans. Another recent review has addressed this topic in mouse models [6].

## Genome-Wide Association Study for Bone or Muscle-Related Phenotypes

**Fig. 1:**
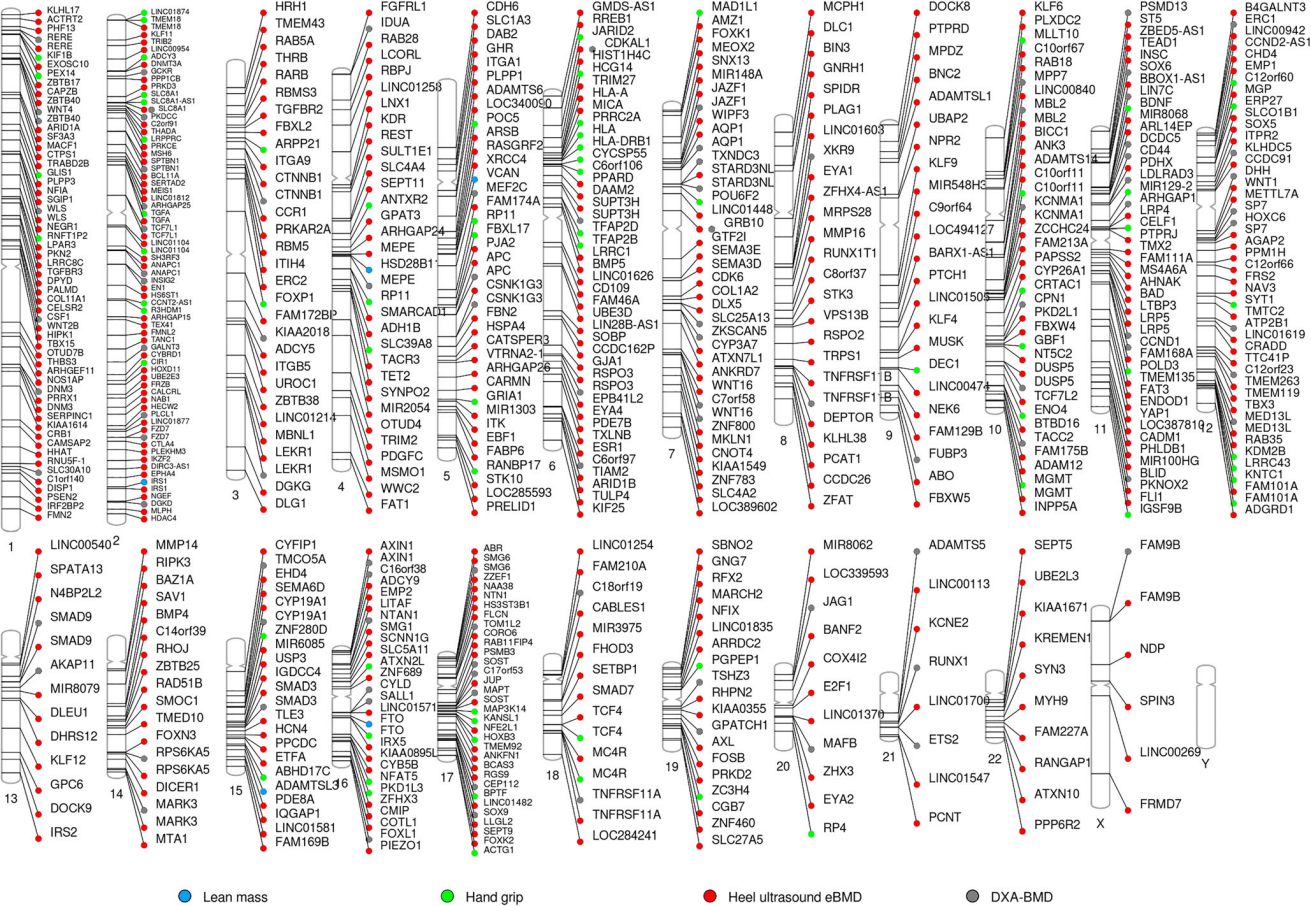
Phenogram showing genome-wide association study results for bone and muscle-related phenotypes. Genes mapping to loci associated with lean mass (light blue), hand grip (light green), heel ultrasound estimated BMD (red), and DXA-derived BMD (gray). The ideogram was constructed using Phenogram http://visualization.ritchielab.psu.edu/phenograms/plot

Risk factors affecting osteoporosis and sarcopenia have a strong genetic component, with heritability estimates above 60% [7]. Genome-wide association studies (GWAS) have identified multiple genetic variants influencing the variability of bone mass (Fig. 1). In total, 62 loci [8–12] have been associated with DXA-derived bone mineral density (BMD) at either the femoral neck or lumbar spine, while 36 loci [13] have been associated with total body BMD. Notably, these GWASs have highlighted known bone-active pathways, i.e., OPG-RANK-RANKL, WNT, and mesenchymal differentiation, among others [14]. One of the greatest successes in the osteoporosis field was achieved through the discovery of the BMD locus harboring *WNT16*, a critical regulator of cortical bone thickness [15] and trabecular bone mass [16]. Moreover, with an ever-growing number of genes discovered by GWASs, novel pathways acting on bone have been identified (e.g., oncogenic and melanogenesis pathways). Recently, 518 loci have been associated with ultrasound-derived heel BMD [17, 18], estimated in more than 400,000 participants of the UK Biobank (UKBB) study. Together, these studies have provided new insights into the pathophysiology of osteoporosis, illustrated by the discovery and functional validation of *GPC6* and *DAAM2*. *GPC6* may serve as novel drug target for osteoporosis, since it encodes glypican, which is involved in cellular growth control and differentiation. Moreover, GPC6 loss of function leads to increased bone mineral content and developmental skeletal abnormalities. *DAAM2* also may be a potential drug target for osteoporosis as it shows effects on bone strength, porosity, and quality in murine models by indirect regulation of the canonical Wnt signaling. *DAAM2* was also expressed in human skeletal muscle [19] (Table 1).

**Table 1:** Bone genes discovered by UK Biobank (and other GWAS) and evidence of their molecular role in the muscle

eBMD gene	Muscle-related trait	Reference
*AHNAK*	Gene expression in human skeletal muscle	Su, Ekman et al. 2015
*AQP1*	Gene expression in human skeletal muscle	Su, Ekman et al. 2015
*ARHGAP26*	Positive/mouse skeletal muscle mass KO	Verbrugge, Schönfelder et al. 2018
*BCKDHB*	Gene expression in human skeletal muscle	Su, Ekman et al. 2015
*DAAM2*	Gene expression in human skeletal muscle	Su, Ekman et al. 2015
*DLEU1*	Gene expression in human skeletal muscle	Su, Ekman et al. 2015
*GRB10*	Negative/mouse skeletal muscle mass KO	Verbrugge, Schönfelder et al. 2018
*HMGA2*	Positive/mouse skeletal muscle mass KO	Verbrugge, Schönfelder et al. 2018
*IGFBP2*	Negative/mouse skeletal muscle mass overexpress	Verbrugge, Schönfelder et al. 2018
*MMP9*	Positive/mouse skeletal muscle mass overexpress	Verbrugge, Schönfelder et al. 2018
*MPP7*	Gene expression in human skeletal muscle	Su, Ekman et al. 2015
*PPARD*	Positive/mouse skeletal muscle mass overexpress	Verbrugge, Schönfelder et al. 2018
*SMAD3*	Positive/ mouse skeletal muscle mass KO	Verbrugge, Schönfelder et al. 2018
*SMAD7*	Positive/mouse skeletal muscle mass KO	Verbrugge, Schönfelder et al. 2018
*SOX6*	Positive/mouse skeletal muscle mass KO	Verbrugge, Schönfelder et al. 2018

*eBMD* estimated bone mineral density, *KO* knock out

In contrast, the fewer number of GWAS of muscle-related phenotype provide less biological insight about the pathways leading to the development of sarcopenia (Fig. 1). To date, only five loci (*HSD17B11*, *VCAN*, *ADAMTSL3*, *IRS1*, and *FTO*) have been robustly associated with lean mass (total and/or appendicular) [20], which constitutes a good proxy for skeletal muscle mass [21]. Three out of the five lean mass-associated SNPs identified by GWAS are significantly enriched in enhancers and promoters acting in muscle cells. Recently, the same study identified TNRV6B as additional lean mass locus after more stringent adjustment for fat [22]. However, the exact biological pathways affecting muscle mass still remain unknown. Two recent grip strength GWAS, a proxy for muscular function, have been more fruitful, yielding 64 muscle strength-related loci [23, 24] identified within the UK Biobank. The loci found associated with grip strength contain genes implicated in the structure and function of skeletal muscle (*ACTG1*), excitation-contraction coupling (*SLC8A1*), or involvement in the regulation of neurotransmission (*SYT1*), which provides additional evidence of the genetic control exerted on this muscle trait [23]. These findings highlight that the grip strength phenotype has a neuromuscular component, since it also characterizes the ability of the peripheral nervous system to appropriately recruit muscle cells. Briefly, Actg1-ms knockout mice display muscle weakness and whole-body functional deficit [25]. *SLC8A1* overexpression in muscle cells induces muscular changes similar to those of muscular dystrophy [26]. Finally, *SYT1* has been linked to synaptic defects at the neuromuscular junctions in mouse model of spinal muscular atrophy [27]. Further, three lead SNPs (rs10186876, rs6687430, and rs754512) for grip strength map in the vicinity of genes implicated in monogenic syndromes characterized by neurological and/or psychomotor impairment like French-Canadian variant of Leigh syndrome characterized (*LRPPRC*), Zellweger Spectrum Peroxisomal Biogenesis Disorder (*PEX14*), and Koolen-de Vries syndrome (*KANSL1*) [23].

## From Cross-Phenotype Effects to Pleiotropy: Bone and Muscle

### Basic Concepts

Multiple genes identified by GWAS of muscle-related traits have also been associated with heel BMD in the UKBB GWAS (Table 2). While such cross-phenotype associations may arise due to biological pleiotropy, there are other reasons that can lead to spurious pleiotropy. Therefore, cross-phenotype associations should not be always regarded as the consequence of true pleiotropy. Pleiotropy commonly refers to a phenomenon in which a genetic locus (a gene or a single variant within a gene) affects more than one trait or disease [28]. It can be classified as (1) biological—when a gene has a direct biological effect on more than one trait or biomarker; (2) mediated—where a gene has a biological effect on one trait which lies on the causal path to another trait and thus the gene affects both traits; and (3) spurious—when different forms of biases can lead to false-positive findings [29]. The most common causes of spurious pleiotropy are ascertainment bias and phenotypic misclassification [29]. The study of pleiotropy may have tremendous clinical implications in the fields of osteoporosis and sarcopenia by discovering new drug targets acting on both muscle and bone.

**Table 2:** Overlapping genes between different bone parameters and different muscle-related traits

Muscle-related trait	Gene	eBMD *P* value	Muscle-related traits *P* value	Reference
Bone mineral density				
Total body lean mass	*MC4R*	2.0 × 10^−15^	1.0 × 10^−18^	Karasik et al. 2019
Total body lean mass	*FTO*	1.6 × 10^−26^	1.4 × 10^−09^	Zillikens et al. 2017
Hand grip strength	*IRS1*	4.7 × 10^−08^	1.5 × 10^−11^	Zillikens et al. 2017
Hand grip strength	*MGMT*	2.3 × 10^−22^	1.0 × 10^−13^	Tikkanen et al. 2018
Hand grip strength	*TCF4*	9.4 × 10^−10^	5.9 × 10^−15^	Tikkanen et al. 2018
Hand grip strength	*TMEM18*	2.0 × 10^−11^	5.4 × 10^−22^	Tikkanen et al. 2018
Hand grip strength	*LINC01104*	7.9 × 10^−11^	3.1 × 10^−09^	Tikkanen et al. 2018
Hand grip strength	*MC4R*	2.0 × 10^−15^	2.1 × 10^−19^	Tikkanen et al. 2018
Hand grip strength	*PEX14*	6.7 × 10^−13^	5.6 × 10^−11^	Willems et al. 2017
Hand grip strength	*SLC8A1*	7.4 × 10^−38^	7.7 × 10^−09^	Willems et al. 2017
Hand grip strength	*TGFA*	9.3 × 10^−19^	4.8 × 10^−13^	Willems et al. 2017
Bivariate analysis with bone strength index				
Appendicular lean mass	*FADS1*		p_b_ = 1.6 × 10^−07^	Han et al. 2012
Bivariate analysis with appendicular bone size				
Appendicular lean mass	*GLYAT*		p_b_ = 1.8 × 10^−06^	Guo et al. 2013

*p*_*b*_ bivariate *p* value for bone/muscle pair, *eBMD* estimated bone mineral density

### Shared Biology: Evidence from Multivariate Analysis

While GWAS are typically performed for the study of one trait at a time, more recently methodological advances have enabled the simultaneous GWAS assessment of multiple traits. In humans, joint analysis of multiple, correlated traits, i.e., multivariate GWAS, has been instrumental to the identification of pleiotropic candidate SNPs/loci associated with traits related to both bone and muscle metabolism. GWAS investigating both bone and muscle phenotypes have produced a list of potential candidate genes for further biological validation such as *PRKCH* and *SCNN1B* [30] in 3844 Europeans; *HK2*, *UMOD*, *MIR873*, and *MIR876* [31] in 1627 unrelated Chinese adults individuals; *HTR1E*, *COL4A2*, *AKAP6*, *SLC2A11*, *RYR3*, and *MEF2C* [32]; and *GLYAT* [33] in 1627 unrelated Chinese adults. In addition, the GWAS–identified *METTL21C* was found to be a novel pleiotropic gene suggestive of association with both muscle and bone acting through the modulation of the NF-κB signaling pathway [34••]. This gene has been implicated in the etiology of inclusion body myositis (skeletal muscle) and of early-onset Paget’s disease (bone) [35]. Subsequent studies confirmed that *METTL21C* polymorphisms contribute to peak bone mass in Chinese males [36]. Moreover, frail subjects showed higher expression levels of METTl21C compared to young healthy older adults [37]. *METTL21C* belongs to the METTL21 family of the methyltransferase superfamily and possesses protein-lysine N-methyltransferase activity [38]; its close homolog, *METTL21D* was found to bind to the chaperone valosin-containing protein (VCP, a.k.a. VCP/p97), known to play a role in a muscle atrophy disease [39]. More recently, Medina-Gomez et al. [40••] performed a bivariate GWAS meta-analysis of total body lean mass and total body less head BMD in 10,414 children. The study identified variants with pleiotropic effects in eight loci, mostly already known for BMD (*WNT4*, *GALNT3*, *MEPE*, *CPED1/WNT16*, *TNFSF11*, *RIN3*, and *PPP6R3/LRP5*), but also the *TOM1L2/SREBF1* locus not previously associated with BMD or lean mass. The protein was highly expressed in mouse calvaria-derived cells during osteoblastogenesis and showed the highest expression peak at the onset of osteoblast mineralization in human mesenchymal stem cells. Moreover, SREBP1 indirectly downregulated several key regulators of myogenesis (i.e., MYOD1, MYOG, MEF2C). Notably, SREBF1 exerted opposite effects on the differentiation of myocytes and osteoblasts, which are in line with the opposite effects observed on BMD and lean mass in the bivariate analysis.

## Human and Animal Bone-Focused Knock-out “Models” Comprising Muscle Phenotypes

Congenital disorders affecting bone or muscle are often associated with deficits in the other tissue as well. For example, reduced muscle capacity and strength have been observed in children with osteogenesis imperfecta (OI) [41, 43], where the primary defect comprises the skeletal system. About 85% of the OI cases are caused by mutations in the *COL1A1* and *COL1A2* genes. These mutations affect the production of the α1/α2 chains of type 1 collagen, an important structural component of the bone, skin, tendons, ligaments, and other connective tissues [44]. Animal studies have also observed muscle weakness in OI mice [45], providing additional evidence for the muscle abnormalities in OI. The exact mechanisms leading to muscle weakness are yet unclear but they can be result of intrinsic muscle factors or direct paracrine effects of the abnormal bone matrix (i.e., increased TGF-β signaling in OI decreases lean mass).

Further, muscle abnormalities have been also noted in individuals with hypophosphatemic rickets; hereditary phosphate wasting disorders commonly caused by point mutations in *PHEX*, *FGF23*, and *DMP1* genes. Children carriers of any of these mutations have soft bones (rickets), growth retardation, poor dental development, and elevated serum FGF23 levels [46]. It has been shown that accumulation of FGF23 is strongly associated with muscle function abnormalities in humans [47, 48]. Additionally, murine models have shown reduced grip strength and impaired muscle forces in the *Hyp* (model of PHEX deficiency) and *Dmp1* null (DMP1 deficiency) mice [49]. However, the underlying mechanisms leading to skeletal muscle abnormalities in individuals with hypophosphatemic rickets have not been characterized.

## Human and Animal Knock-out Models of Muscle Comprising Bone Phenotypes

Disorders of muscle often present with bone abnormalities. For example, in Duchenne muscular dystrophy (DMD) and Becker muscular dystrophy, the primary defect leading to disease pathogenesis is degeneration of striated muscle. The mutations in the *DMD* gene encode the dystrophin protein, causing Duchenne and Becker muscular dystrophies. Yet, impairment of bone health in the form of low BMD and increased incidence of bone fractures are well-recognized clinical components of the DMD phenotype [50–52]. The deleterious effects of DMD on bone can be mediated by several mechanisms. These include downstream functional effects on bone involving the nuclear factor of κB pathway and cytokine-mediated (IL-6) activation of osteopontin (OPN); disturbances of calcium metabolism; deterioration of biomechanical stimuli with disease progression, side effects of corticosteroid treatment and many comorbidity processes derived from the disease. Also, other single-gene disorders including those derived from mutations in *MTM1*, *RYR1*, and *DNM2* have been implicated in the alteration of skeletal muscles. These congenital myopathies are characterized by an alteration in the contractile apparatus of the muscle (myofibers) followed by loss of muscle mass, increased muscle weakness, and decrease in bone mass. However, due to the complex interplay between muscle and bone, it is not clear if these changes in bone mass are result of the decreased mechanical loading, the muscular paracrine effect, the shared biology, or combination of all of these factors.

Another example of a potentially pleiotropic gene is the myostatin (*MSTN*, a.k.a. growth and differentiation factor 8, *GDF8*) gene, a member of the TGF-β superfamily, which is secreted primarily by the skeletal muscle cells [53]. Point mutations in *MSTN* lead to decreased production of functional myostatin causing muscle hypertrophy in humans and animals [54]. Recently, the relationship between myostatin and bone has been actively investigated in animal models. *MSTN* depletion leads to increased BMD and strength through many different pathways. First and foremost, the loss of myostatin is followed by doubling of the skeletal muscle mass, which increases mechanical loading on bone. However, the increase in lean mass can also have indirect positive effects on the bone by increasing IGF-1 levels [55]. Such studies have found that inhibition of the myostatin pathway increases proliferation and differentiation of osteoprogenitor cells and leads to bone mass accrual [56]. Further, it has been shown that haploinsufficiency of myostatin protects against aging-related declines in muscle function and enhances the longevity of mice [57]. This suggests that, beyond a known effect of myostatin on bone [58], there is also a systemic effect of this gene.

Further, myostatin binds to the soluble activin type IIB receptor (ACVR2B) which forms an activin receptor complex with activin type 1 serine/threonine kinase receptors (ACVRs). Loss of function of activin type I receptor (ACVR1) in osteoblasts increases bone mass and activates canonical Wnt signaling through suppression of the Wnt inhibitors SOST and DKK1 [59••]. Recently, a study has also shown that myostatin inhibits osteoblastic differentiation by suppressing osteocyte-derived exosomal microRNA-218, suggesting a possible novel mechanism in the bone-muscle crosstalk [60].

Myocyte enhancer factor-2 (MEF2C) is a member of the MADS-box superfamily of transcriptional regulatory proteins relevant for skeletal muscle development, sarcomeric gene expression, and fiber type control. MEF2C directly regulates myomesin gene transcription and loss of Mef2c in skeletal muscle results in improper sarcomere organization and disorganized myofibers. Recent studies have found that a super activating form of MEF2C causes precocious chondrocyte hypertrophy, ossification of growth plates, and dwarfism [61]. *Mef2c*^*+/−*^ presented lack of ossification within the sternum. *Mef2c*^*loxP/KO*^*; Twist2-Cre* mice had shortened limbs from birth [61]. Moreover, the MEF2 activity is enhanced by the increase in mitogen-activated protein kinases (MAPKs) and decrease in histone deacetylases (HDACs). HDAC inhibitors have been tested in a muscular dystrophy model in mice which promoted the formation of muscles with increased cross-sectional area [62]. Interestingly, HDAC5 part of the HDAC family is a known BMD locus [8].

Last but not least, GWAS studies have identified variants in *FAM210A* as strongly associated with fracture risk but less strongly with BMD. Moreover, SNPs near *FAM210A* were nominally (*p* < 0.05) associated with lean mass in adults [1]. Interestingly, a recent study in mice has shown that *Fam210a* was expressed in muscle mitochondria and cytoplasm but not in bone [1]. Notably, grip strength and limb lean mass were reduced in both tamoxifen-inducible *Fam210a* homozygous global knockout mice (*TFam210a*^*−/−*^) and skeletal muscle cell-specific knockout mice (*Fam210aMus*^*−/−*^). Moreover, microarray analysis showed decreased levels of *Myog* and *Chdh15*, transcription factors relevant for myoblast differentiation, and terminal muscle differentiation, respectively. Also, decreased BMD, bone biomechanical strength and bone formation, and elevated osteoclast activity were observed in *TFam210a*^*−/−*^ mice [1]. Furthermore, the authors showed that *Mmp12* was increased in muscle cells of *TFam210aMus*^*−/−*^ mice, which can enhance osteoclast function in bone. Therefore, *Fam210a*, while being expressed in muscle, plays an influential role on bone quality and quantity.

## Muscle and Bone: Beyond Biomechanics

Multiple metabolic communications have been identified between bone and muscle in humans and rodents. There are numerous indications that the muscle “secretome” contains *osteoinducer* and *osteoinhibitor* myokines [63]; it also seems likely that bone cells secrete myoinducer and myoinhibitor osteokines [64]. The skeletal muscle secretome accounts for various molecules that affect bone including insulin-like growth factor-1 (IGF1), fibroblast growth factor (FGF2), interleukins (IL6, IL15), myostatin, osteoglycin, osteoactivin, and others (reviewed by [64]). Even though studies on the potential effects of bone on muscle metabolism are still sparse, a few osteokines have been identified. Prostaglandin E2 (PGE2) and WNT family member 3A (*WNT3A*), which are secreted by osteocytes, are thought to impact skeletal muscle cells. Interestingly, *WNT3A* and several other WNT factors have been identified in GWAS of BMD. Also, osteocalcin and IGF-1, which are produced by osteoblasts, and sclerostin, which is secreted by osteocytes and osteoblasts, exert effects on muscle cells. Further, bones and muscles are controlled by mitochondrial genetics that standard GWAS cannot reliably scrutinize given the sparse number of mitochondrial markers on genotyping arrays and the difficulties to quantifying mitochondrial heteroplasmy. Previous studies have revealed the critical role of mitochondria in the differentiation of multiple cell types, including cardiomyocytes [65] and myoblasts [66]. Additionally, osteoporosis and sarcopenia seem to be more prevalent among patients with mitochondrial disorders. Thus, refined GWAS efforts can help understand the underlying mechanisms of the mitochondria, using intensity-based assessments of mitochondrial copy number. In addition to the mitochondrial metabolism, the genes controlling overall energy metabolism might also exert an impact on bone and muscle. Interestingly, skeletal muscle-specific disruption of the circadian rhythms by *Bmal1* deletion has been shown to disrupt skeletal muscle metabolism [67], whereas BMAL1 deficiency results in a low bone mass phenotype [68]. However, this gene has not yet been identified by any GWAS on BMD.

## The Potential of Genetic Discoveries to Guide Drug Target Identification

Incorporating genetic information in the drug discovery process can improve disease-specific drug target identification and validation. Combining drug mechanisms with genetic mechanistic information increases the success in drug discovery over approaches that do not include genetic information, especially for drug targets related to musculoskeletal (BMD), metabolic, and blood traits [69].

From the molecular factors discussed above, two have been followed as potential drug targets. It has been well established that myostatin is a negative regulator of muscle and bone mass. Therefore, there is hope that studies into myostatin inhibitors may have therapeutic application in treating muscle-wasting diseases such as muscular dystrophy. Bialek et al. [58] have investigated the role of myostatin by administrating myostatin neutralizing antibody (Mstn-mAb) or soluble myostatin decoy receptor (ActRIIB-Fc) in young adult mice. Interestingly, while both antibodies increased muscle mass, only ActRIIB-Fc also increased bone mass. Thus, a therapeutic agent that has this dual effect represents a potential approach for the simultaneous treatment of osteoporosis and or sarcopenia. Another potential target is FGF23. Currently, clinical trials of neutralizing anti-FGF23 antibody for patients with FGF23-related hypophosphatemic diseases are ongoing. First of all, FGF23 production is stimulated through signaling acting through the FGF receptor. It has also been shown that repeated administration of FGF receptor inhibitors causes increased bone growth and mineralization in *Hyp* mice [70]. Similarly, weekly injection of FGF23 antibodies increased BMD in *Hyp* mouse, while with a higher dose, there was also an increase in grip strength. To note, in a phase I clinical trial, administration of various amounts of anti-FGF23 antibodies increased tubular maximum transport of phosphate per glomerular filtration rate (TmP/GFR) in adult patients with X-linked hypophosphatemia (XLH) [71, 72]. Nevertheless, it needs to be tested if anti-FGF23 antibody can improve or cure rickets/osteomalacia or their clinical presentations such as bone pain and muscle weakness. Although the clinical implications of these findings are still far-reaching, both examples illustrate the diverse opportunities for the characterization of drug targets that can prevent muscle and bone abnormalities.

The approach is not free of limitations, as genetically derived targets may also have undesired secondary effects. For example, MEF2C has been suggested as novel drug target for therapeutically enhancing muscle performance. Targeting these genes may have a significant impact on the treatment of muscle. However, MEF2C also is related with pathological cardiac hypertrophy. Hence, off-target effects can be considerable. The key will be to improve muscle performance and prevent cardiotoxicity at the same time when targeting these genes [73]. Either way, this also illustrates the potential of the genetic evidence underlying drug targets to typify the presence of adverse effects before embarking on expensive experimentation.

## Summary and Future Directions

Overall, there are many evolutionary, biological, and clinical factors that couple the pathogenesis of sarcopenia and osteoporosis. Both muscle and bone also act as endocrine organs [74, 74] and share common genetic influences. Although challenging, there is a growing research enterprise aimed at elucidating and unraveling new mechanisms of muscle-bone crosstalk. Many questions still remain unanswered and need to be addressed through the integration of in vitro and in vivo models. For example, what are the exact mechanisms underlying the cross-organ effects? Do muscle factors, by stimulating bone metabolism, also lead to increased release of myoinducer and myoinhibitor osteokines? More importantly, the question remains as how the aging process influences muscle and bone metabolism, including the underlying molecular factors. Further, the role of central mechanisms in co-regulation of the musculoskeletal system needs to be investigated in its entirety rather than its parts. Finally, the study of pleiotropy may provide novel insights into disease pathophysiology with the potential of leading to the identification of drug targets that simultaneously prevent or treat both diseases.
